# Can Any Drug Be Repurposed for Cancer Treatment? A Systematic Assessment of the Scientific Literature

**DOI:** 10.3390/cancers13246236

**Published:** 2021-12-13

**Authors:** Nicolai Stransky, Peter Ruth, Matthias Schwab, Markus W. Löffler

**Affiliations:** 1 Department of Pharmacology, Toxicology and Clinical Pharmacy, Institute of Pharmacy, University of Tübingen, Auf der Morgenstelle 8, 72076 Tübingen, Germany; peter.ruth@uni-tuebingen.de; 2 Department of Radiation Oncology, University Hospital Tübingen, Hoppe-Seyler-Str. 3, 72076 Tübingen, Germany; 3 Dr. Margarete Fischer-Bosch Institute of Clinical Pharmacology, Auerbachstr. 112, 70376 Stuttgart, Germany; Matthias.Schwab@ikp-stuttgart.de; 4 Department of Clinical Pharmacology, University Hospital Tübingen, Auf der Morgenstelle 8, 72076 Tübingen, Germany; 5 Cluster of Excellence iFIT (EXC2180) ‘Image-Guided and Functionally Instructed Tumor Therapies’, Faculty of Medicine, University of Tübingen, 72076 Tübingen, Germany; 6 German Cancer Consortium (DKTK), Partner Site Tübingen, 72076 Tübingen, Germany; 7 Department of General, Visceral and Transplant Surgery, University Hospital Tübingen, Hoppe-Seyler-Str. 3, 72076 Tübingen, Germany; 8 Department of Immunology, Interfaculty Institute for Cell Biology, University of Tübingen, Auf der Morgenstelle 15, 72076 Tübingen, Germany

**Keywords:** repurposing, repositioning, cancer, systematic assessment, meta research

## Abstract

**Simple Summary:**

Drug repurposing strategies utilize drugs, already approved by regulatory authorities, to test their efficacy against different diseases. While this approach is increasingly used, according to the literature, there are few systematic assessments of these efforts so far. In this work, we tried to answer the question: How many approved drugs show anti-cancer effects according to the literature? We found that the majority (69%) of the approved drugs we analyzed did show anti-cancer effects in preclinical studies. The assessment of the methodological quality of the reports, namely, the reporting quality and usage of bias-reducing methods, showed that the methodological quality of the articles was by and large moderate, while many items of the quality assessment were lacking in most reports (for example, blinding, preregistration, power calculations, and detailed information on lab animals). We hypothesize that the current reward systems favor positive results over high methodological quality, probably leading to many false-positive results.

**Abstract:**

Drug repurposing is a complementary pathway for introducing new drugs against cancer. Broad systematic assessments of ongoing repurposing efforts in oncology are lacking, but may be helpful to critically appraise current and future efforts. Hence, we conducted a systematic PubMed search encompassing 100 frequently prescribed and 100 randomly selected drugs, and assessed the published preclinical anti-cancer effects. Furthermore, we evaluated all the identified original articles for methodological quality. We found reports indicating anti-cancer effects for 138/200 drugs, especially among frequently prescribed drugs (81/100). Most were reports suggesting single-agent activity of the drugs (61%). Basic information, such as the cell line used or control treatments utilized, were reported consistently, while more detailed information (e.g., excluded data) was mostly missing. The majority (56%) of in vivo studies reported randomizing animals, while only few articles stated that the experiments were conducted in a blinded fashion. In conclusion, we found promising reports of anti-cancer effects for the majority of the assessed drugs, but speculate that many of them are false-positive findings. Reward systems should be adjusted to encourage the widespread usage of high reporting quality and bias-reducing methodologies, aiming to decrease the rate of false-positive results, and thereby increasing the trust in the findings.

## 1. Introduction

Drug development faces numerous challenges. In oncology, most drugs entering clinical trials will eventually fail to receive regulatory approval (i.e., 14 out of 15 fail) [1]. Across all the drugs approved for solid cancers between 2002 and 2014 by the Federal Drug Administration (FDA), the median overall survival benefit was merely 2.1 months [2]. Despite a steadily widening scope in cancer research, encompassing, e.g., immunotherapy, elucidation of new cancer cell dependencies, and targeting the tumor microenvironment [3], the prognosis for many cancer entities remains poor; this is reflected by the dismal 5-year overall survival rates, e.g., ~5% in glioblastoma [4], ~10% in pancreatic cancer [5], and ~21% in lung cancer [5].

**Figure 1:**
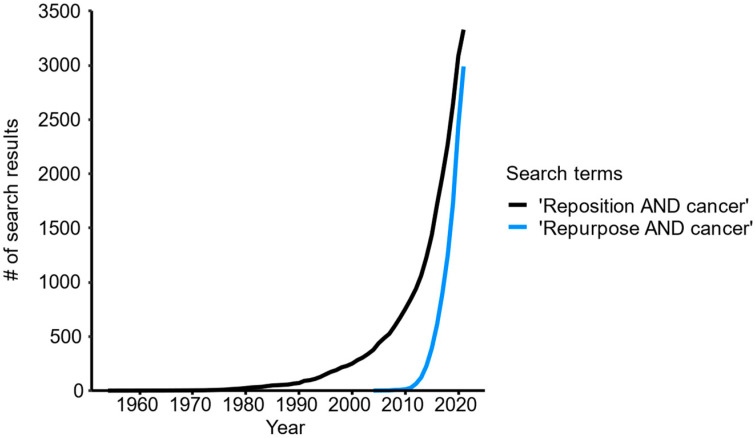
Total number (#) of publications identified in PubMed (https://pubmed.ncbi.nlm.nih.gov, accessed on 13 July 2021) with search terms “reposition AND cancer” or “repurpose AND cancer”.

Drug repurposing, also known as drug repositioning, has gained traction as a potential complementary pathway for introducing new treatments in oncology [6] (see Figure 1), utilizing already approved drugs for non-cancer indications against specific cancer entities.

The strategy holds several potential benefits, which are as follows: Since the drug has already been clinically developed, early development may be shortened. Further, due to an already established use in patients, the safety profile can be well appraised, probably reducing the number of failures in early stage development, due to safety signals [7,8], even though the adverse event profiles may differ in a different patient population or with a different dosing scheme. This may result in a shorter development period, translating to lower costs. A decrease in respective costs, from USD 2–3 billion to USD 300 million, and a shortened development time, from 13–15 years to 6 years, have been estimated for this scenario [9], particularly related to preclinical studies and phase 1 and 2 clinical trials. Drug repurposing may also facilitate precision medicine; for example, the overexpression of COX-2 in human breast cancer tissue [10,11] may be amenable for treatment with celecoxib, a selective COX-2 inhibitor introduced as an analgesic. Further, since targeting multiple pathways with repurposed drugs might prove effective, the tolerability of combined drug regimens may be derived from other patient groups. Real-world observations of beneficial associations, using electronic health records and clinical trial data, are currently driving the intensified repurposing efforts, potentially elucidating new cancer cell dependencies and targets [7]. Another approach is based on the extraction of genes from genome-wide association studies (GWAS) related to traits and/or diseases, to identify novel targets, which may be druggable or can be addressed with biologicals [12].

However, there are also several limitations of the drug repurposing concept, demonstrated by failures in the late-stage development of repositioned drugs [8]. Some authors assume that most repurposed drugs lack single-agent activity and merely increase the efficacy of already existing therapies [13]. While such multi-pronged approaches seem intuitive, critics caution against widespread testing of agents lacking single-agent activity in early stage development, since the pre-test probability for substantial benefits is low. Therefore, abandoning agents without single-agent-activity in early clinical development is recommended [14]. Another problem arising in the context of repurposed drugs is the lower potential revenue, due to shortened or expired patent protection [15]. This contrasts the benefits of confined development expenses, since proving efficacy still requires large and costly clinical trials, usually exceeding the funding available to academic institutions. Furthermore, observational data supporting specific drugs for repurposing suffer from several structural weaknesses, including immortal-time bias [16], selection bias, or the vibration of effects [17]. Hence, many hypotheses will likely fail to confer stable benefits for patients, diluting the benefit of reduced development costs. Finally, it is dangerous to promote drugs approved for other indications, due to easy access and off-label prescribing, which may result in patients demanding access to drugs with insufficient evidence [18], as has previously occurred with methadone [19].

While reviews summarizing the findings from repurposing efforts in oncology are available for specific drugs [6], a systematic assessment of how many approved drugs are actually implicated with anti-cancer effects is lacking, according to our own research.

Here, we report the results of a systematic search in PubMed, identifying preclinical reports of anti-cancer effects for the majority of the assessed drugs. These findings may help readers to contextualize and critically appraise current and future repurposing efforts.

## 2. Methods

**Figure 2:**
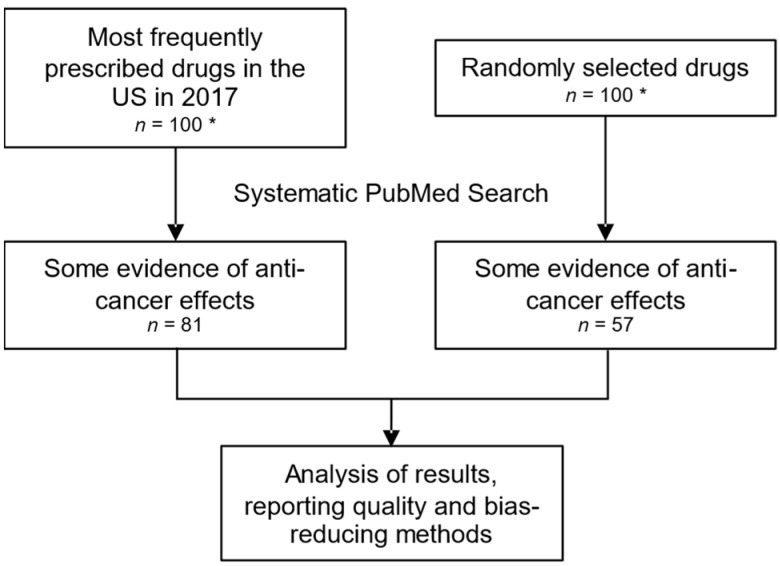
Flowchart of drug selection and subsequent analyses. * All drugs that could be classified as a non-small molecule, mineral, vitamin or endogenous hormone, and all drugs that were already approved for any cancer entity, were excluded.

We systematically searched PubMed (https://pubmed.ncbi.nlm.nih.gov, accessed between 1 September 2020 and 31 December 2020) for reports of anti-cancer drug effects of 200 approved pharmaceuticals and assessed the results, reporting quality and bias-reducing methods in identified articles (see Figure 2). Through this attempt we aim to provide an overview of the landscape of drug repurposing efforts in oncology.

### 2.1. Compilation of Drugs

In order to obtain a more representative sample of drugs, both a set of frequently prescribed drugs as well as a set of randomly selected drugs were compiled. Most frequently prescribed drugs in the year 2017 in the US were extracted from https://clincalc.com/DrugStats/Top300Drugs.aspx (accessed on 1 September 2020). Randomly selected drugs were randomly picked from all FDA-approved drugs (https://www.accessdata.fda.gov/scripts/cder/daf/, accessed on 1 September 2020). Drugs were excluded if they could be classified as a non-small molecule, mineral, vitamin or endogenous hormone, or if the drug was already approved for any cancer entity (in this regard we excluded methotrexate, prednisone and levothyroxine among the most frequently prescribed drugs). Enantiomers were included as their racemic mixture (e.g., citalopram was selected instead of escitalopram). If a drug for the randomly selected drug list was also present among the most frequently prescribed drugs, we replaced it with another random drug to avoid overlap. In this way we selected 100 drugs per group.

### 2.2. Search Strategy and Number of Drugs Implicated with Anti-Cancer Effects

The following three search strategies were utilized in PubMed: The first search strategy ((name of drug) AND ((repurpose AND cancer) OR (reposition AND cancer))) aimed at identifying drugs already being discussed to have repurposing potential in the scientific literature; for example, the first search strategy for acitretin was acitretin AND ((repurpose AND cancer) OR (reposition AND cancer)).

The second search strategy ((name of drug) AND cancer) was intended to identify a broader range of articles. After analyzing all 200 drugs, we utilized a third search strategy ((name of drug) AND cancer proliferation) for all remaining drugs without any hits in the previous searches. The first 20 articles identified by each search strategy were screened for information regarding preclinical anti-cancer effects and all results and methodologies were summarized after discussion and agreement by two reviewers. In general, only information from the article with the most relevant findings, defined by our categorization efforts described below, was extracted and used for the subsequent assessment of methodological quality.

### 2.3. Categorizing Findings of the Studies

Articles were categorized in the following order, which we believe strongly supports future translational potential of respective drugs: Highest priority was given to reviews summarizing the preclinical effectiveness of a drug against cancer. Furthermore, we assigned the same level of evidence to drugs already being tested in clinical trials, since we assumed institutional review board approval for clinical studies should have included a summary of preclinical evidence. Next highest priority was given to drugs with established single-agent activity in (i) in vivo models and (ii) in vitro models. Results describing anti-cancer effects when combined with other drugs were assigned a lower priority and again subdivided into (i) in vivo studies and (ii) in vitro studies. Ultimately, biological plausibility, such as inhibition of a driver mutation, was ranked with lowest priority.

### 2.4. Assessment of the Methodological Quality

All identified original articles were assessed for items indicating high methodological quality, i.e., high reporting standards and the use of methods to reduce bias. In general, we assessed the methods section and searched the whole report for specific terms indicating methodological aspects or reporting quality (see all criteria and a full list of search terms for each item in Tables S1 and S2). For in vitro studies we assessed whether the authors reported the respective cell line, the drug dosage, duration of exposure and solvent of the drug used, whether authors specified exclusion criteria and whether experiments were preregistered and conducted in a blinded fashion. In vivo studies were analyzed regarding information about the species and baseline characteristics of the animals (age, sex and weight), dosing of the drug, route of administration, whether or not the control conditions were specified, adverse events and specification of exclusion criteria. Furthermore, the reports were searched for information about preregistration of the experiments, randomization of animals, blinding of experiments and power calculations.

## 3. Results

Overall, our systematic search identified articles for 138/200 drugs (69%) that implied some activity against cancer. While, amongst the 100 most frequently prescribed drugs, reports of anti-cancer effects were found for 81 pharmaceuticals, we could identify reports showing anti-cancer effects for 57 drugs in the randomly selected group (see Table 1). The first two search strategies identified the majority of reports indicating anti-cancer effects (125/138), whereas strategy 3, which was applied in the remainder of the drugs, identified reports of anti-cancer activity for only 13 drugs, mainly used as supportive treatments in cancer patients.

**Figure 3:**
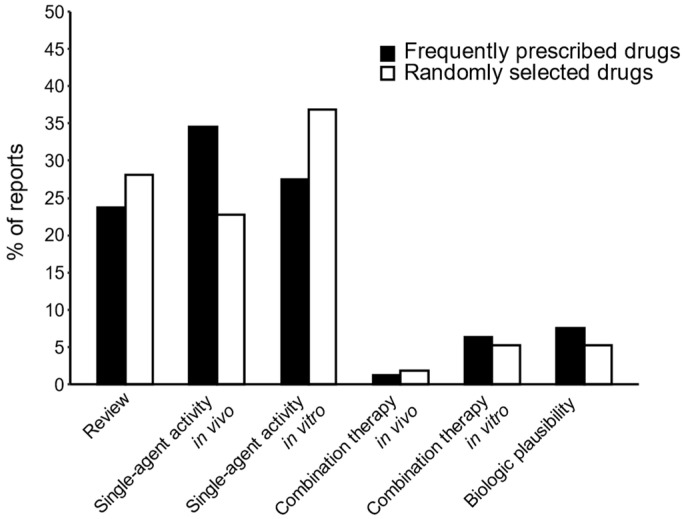
Categorization of identified articles subdivided into frequently prescribed drugs and randomly selected drugs.

Our search and categorization efforts revealed that the vast majority of articles identified were reviews (35/138 drugs, 25%; see Figure 3), or described preclinical single-agent activity (84/138 drugs, 61%). When comparing the identified articles of the most frequently prescribed drugs with randomly selected drugs, the differences between the relative proportions of each category were small, suggesting that the general composition of the articles was comparable between the two selected sets of drugs.

We also compared our search results with the ReDO database (https://www.anticancerfund.org/en/redo-db, accessed on 5 November 2020), a database listing drugs with potential anti-cancer effects that are already approved for other indications. While our search strategy identified all but one drug also contained in the database, we could identify an additional 59 drugs reported to have anti-cancer effects (26 drugs among the most frequently prescribed drugs and 33 among the randomly selected drugs).

**Figure 4:**
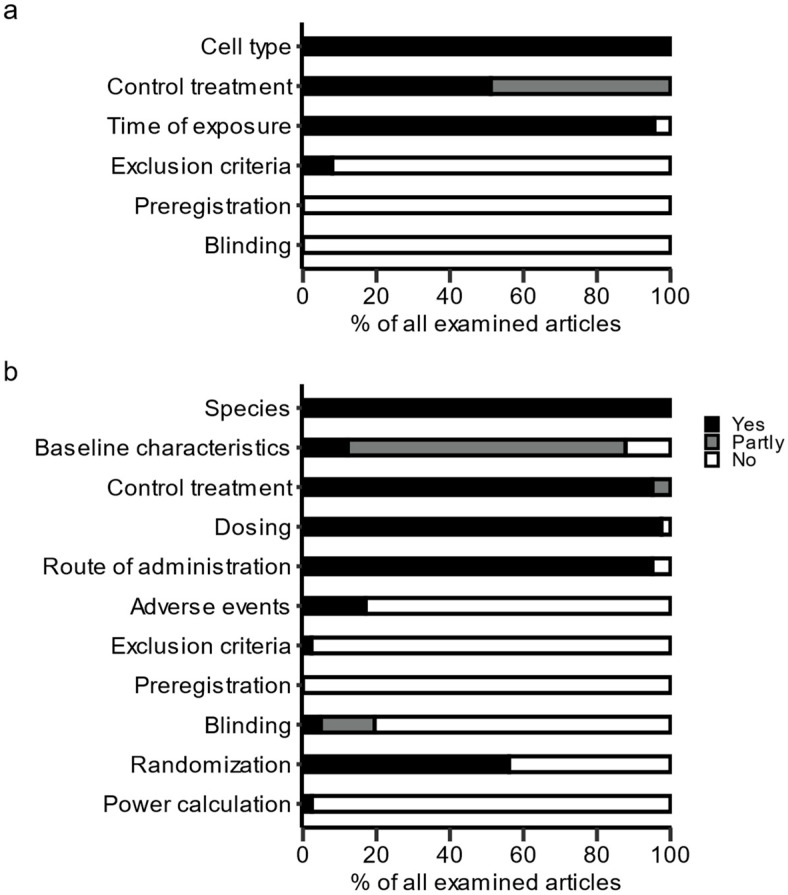
Methodological quality of original articles: (**a**) in vitro studies (*n* = 50) and (**b**) in vivo studies (*n* = 41). Depicted are percentages of articles reporting or applying the respective item.

To address the methodological quality, we assessed all 103 articles with original data identified by our search strategies, except for 9 reports, which were unavailable, and 3 articles, which did not present novel data. In total, we identified 50 articles that provided in vitro or in silico data, and 41 in vivo studies. The assessments of reporting quality and bias-reducing methods are shown in Figure 4.

All the assessed in vitro studies reported the cell line used for the experiments. Furthermore, all the publications reported using a control; however, 49% of the studies did not explicitly mention the diluent used to dissolve the drugs. The duration of drug exposure was reported in most of the publications (96%). The authors of in vivo studies reported the species of the animals in every article, and 76% provided some baseline characteristics. However, only 11% of the assessed articles reported precise baseline characteristics of the animals, including the age, weight and sex of the animals. In addition, adverse events were only mentioned in 17% of the assessed articles. Disclosure of exclusion criteria or mentioning excluded data were the exceptions, and were only reported in 8% of the in vitro studies and 2% of the in vivo studies.

Bias-reducing methods mostly consisted of the randomization of animals for in vivo studies; 56% of the publications reported randomizing the animals to different treatment arms. None of the in vitro studies reported blinding of experiments, whereas 5% of the in vivo studies consistently used blinding and 15% blinded some of their experiments. Only one article (2%) reported a power calculation to determine the required sample size of the planned animal experiments, and none of the 91 analyzed articles stated that the study had been preregistered.

All the relevant raw data are provided in the Supplementary Materials of this article (Table S3).

## 4. Discussion

This study originated from the simple assumption that there are probably published reports of anti-cancer effects for most of the frequently prescribed drugs. We reasoned that, if found to be true, this might cast some critical light on drug repurposing efforts in general, since it is not conceivable that nearly all drugs actually do work against cancer. Instead, this finding might support some of the widespread criticism of biomedical research, which argues that many published results are probably false-positives [20].

Our findings show that the scientific literature actually contains findings of anti-cancer effects for the majority of the assessed drugs. As expected, anti-cancer effects were reported more often for frequently prescribed drugs than for randomly selected drugs, speculatively due to easier access, lower purchasing costs, or well-known pharmacokinetics and pharmacodynamics.

After assessing all 200 drugs with our first two search strategies, we recognized the following pattern: our search strategies were too simple to also account for drugs that are used as supportive treatments in cancer patients, and we primarily identified articles dealing with their original use; for example, our search strategies yielded several articles regarding gabapentin’s analgesic effects in cancer patients. Hence, we additionally searched the remaining drugs that did not produce any hits in the previous searches with a third search strategy, ((name of drug) AND cancer proliferation), identifying another 13 drugs with putative anti-cancer effects. This supports the notion that more nuanced search strategies might have identified even more drugs. To further add to this point, we used three additional search strategies after finalizing the manuscript and identified another six drugs with anti-cancer activity according to the literature (see the results in the Supplementary Materials).

While single-agent activity may be useful for prioritizing drugs in the clinical setting, our results indicate that most of the drugs implicated with anti-cancer properties show single-agent activity (84/138 drugs, 61%). Hence, suggesting trialists to focus on drugs with single-agent activity in preclinical studies may be futile for the prioritization of promising drug candidates.

We classified the methodological quality of the analyzed articles as moderate. Basic information about the conducted experiments, such as animal species, cell lines, or control treatments used, was consistently reported, whereas more detailed information, e.g., about the exclusion of data, precise baseline characteristics of the animals, or adverse events, was missing in the majority of the assessed articles. Such information may be relevant to replicate the findings and to gain more trust in the results. We found higher ratios of randomization in the in vivo studies (56%) as compared to the findings of other authors, who found randomization rates of 4% [21] up to 33% [22]. In contrast, the blinding of experiments remained the exception. Previous findings suggest that nonblinded experiments may lead to more significant findings and higher effect sizes compared to blinded experiments [23]. Only one study reported using a power calculation according to our research, while other authors identified mostly low-powered studies in preclinical research [24], which may lead to even more false-positive findings [25]. Finally, none of the articles mentioned a preregistration of experiments, which is an effective method to reduce selective reporting of results and statistical flexibility [26]. Two recent studies reported that only about two thirds of all animal experiments are eventually published or presented at conferences, even after long follow-up periods [27,28], potentially skewing the literature even more towards significant results.

Given these findings, we question that the majority of approved drugs can actually be repurposed against cancer, and, therefore, we speculate that a relevant proportion of these findings may likely constitute false-positives [29,30]. In contrast to the many positive findings in preclinical research, there are very few examples of successfully repurposed drugs in oncology. The most prominent example is thalidomide, which was first clinically tested in multiple myeloma patients more than 20 years ago [31,32]. Another example is the selective estrogen receptor modulator raloxifene, which was approved by the FDA in 2007, to reduce the risk of invasive breast cancer in postmenopausal women at high risk [33], once again long before most systematic repurposing efforts were even started (see Figure 1).

This current situation may be fueled by reward systems emphasizing traditional promotion criteria [34], favoring selective reporting of ‘significant’ findings and the utilization of permissive research practices. It has been suggested that this bias might be remedied by several methods [35], many of which are already common practice in clinical research, including preregistration of experiments, more detailed reporting of experiments, reporting of negative studies, blinding of experiments, randomization of animals, powering animal studies adequately, and independent replication efforts. Reward systems should be adjusted to account for several of these non-traditional items to foster higher methodological standards in preclinical science, potentially reducing the number of false-positive findings reported, and, hence, making drug repurposing efforts more reliable and efficient.

This work has several limitations. Randomly selected drugs were influenced by redundancy, e.g., by different drug formulations or trade names present in the FDA drug list, potentially favoring more common drugs over rather exotic ones; for example, the list contained different formulations or trade names of phenytoin, which appeared at least five times, while diatrizoate was only present twice. Secondly, our search strategy was simple and cannot claim comprehensiveness, conceivably biasing our findings towards lower percentages of potentially repurposable drugs against cancer. Thirdly, it was beyond the scope of this work to critically appraise the quality of each study in detail, and we only reported the results as published by the respective authors. Lastly, in determining the methodological quality of the original articles, some articles may have been misclassified, e.g., when the items were reported in unusual ways and, thus, not adequately identified by our assessment.

## 5. Conclusions

Our results support the notion that the scientific literature contains reports of anti-cancer effects for a large portion of approved drugs, particularly those frequently prescribed. The possibility that all of these drugs actually work against some kind of cancer cannot be excluded; however, this is a rather unlikely scenario. While overly optimistic analyses of not-yet approved drugs are dangerous in themselves, this is aggravated for approved drugs. Public media may hype repurposable drugs based on unreliable findings in preclinical studies, leading to patients demanding off-label prescriptions. Researchers evaluating the repurposing potential of approved drugs should thus apply particularly high standards and methodological rigor to avoid bias that might impact the trust in physicians, and in science itself. Changes in reward systems may be considered as a potential solution, since they could lead to changes in this regard.

## Figures and Tables

**Table 1:** Results of the systematic search.

Systematic Search		Frequently Prescribed Drugs	Randomly Selected Drugs
**Drugs reported to have anti-cancer effects**			
Identified by all search strategies		81	57
Identified by search strategy 1		42	29
Identified by search strategy 2		30	24
Identified by search strategy 3		9	4
**Categorization of findings**			
Review		19	16
Single-agent activity	in vivo	28	13
	in vitro	22	21
Combination therapy	in vivo	1	1
	in vitro	5	3
Biological plausibility		6	3

## Supplementary Materials

The following are available online at https://www.mdpi.com/article/10.3390/cancers13246236/s1: Table S1: Reporting quality; Table S2: Methods to reduce bias; Table S3: Raw data.
