# Supplementary material for: Can Any Drug Be Repurposed for Cancer Treatment? A Systematic Assessment of the Scientific Literature
Source: Cancers (Basel). 2021 Dec 13;13(24):6236. doi: 10.3390/cancers13246236 (PMC8699650; doi:10.3390/cancers13246236)
Supplement: Supplementary file 1 [file cancers-13-06236-s001.zip › cancers-1456605-final-supp/cancers-1456605-supp-final-updated.pdf]

Supplementary Materials

Can Any Drug Be Repurposed for Cancer Treatment? A Systematic Assessment of the Scientific Literature

Nicolai Stransky, Peter Ruth, Matthias Schwab and Markus W. Löffler

1. Additional search strategies requested after finalizing the manuscript

To follow-up our claim that additional search strategies would probably have identified even more drugs reported to have anti-cancer effects, we conducted three additional search strategies after the manuscript was finalized.

We searched all drugs, for which we did not identify any anti-cancer findings with our previous search strategies, with 3 additional search strategies according to the methods already described in our manuscript.

- (Name of Drug) AND anticancer
- (Name of Drug) AND (cancer progression)
- (Name of Drug) AND antiproliferative

In total, we identified reports of anti-cancer effects for another 6 drugs, namely Buprenorphine, Cefotaxime, Lubiprostone, Nandrolone, Amoxicillin and Rivaroxaban. Four reports mentioned single-agent activity in vitro and one report in vivo activity. In another study rivaroxaban was tested in a clinical trial for antiproliferative activities [1].

Thus, according to our hypothesis in the manuscript our assessment likely underestimates the actual number of drugs with alleged anti-cancer effects described in the literature to a small extent.

2. Assessing methodological quality of results – search strategies and search terms

Table S1 and S2 show our approach to assess the articles for methodological quality, i.e., reporting quality (Table S1) and methods to reduce bias (Table S2). For several items we combined analyzing the method section with additionally searching the whole report for specific search terms. For some items we only analyzed the method section or only searched the report for specific search terms. Many search terms are abbreviations to account for different word endings. For example, "dissolv" was used to capture, among others, dissolved, dissolve and dissolving. Furthermore, the tables list criteria that led to the definition of 'partly' reporting respective items or 'partly' applying methods to reduce bias.

Table S1. Reporting quality.

Item	Search Strategy	Grading
Cell type	Analyze method section	Yes or No
Control treatment	Analyze method section<br>Additional search terms:<br>• Dmso<br>• Dissolv<br>• Solven	Yes<br>Partly: solvent not specified<br>No
Time of exposure	Analyze method section	Yes or No
Exclusion criteria or excluded data points	Search terms<br>• "Excl"	Yes or No
Species of animals	Analyze method section	Yes or No
Baseline characteristics (age, weight and sex of animals)	Analyze method section<br>Additional search terms<br>• "Charact"<br>• "Age"<br>• "Sex"<br>• "Male"	Yes<br>Partly: did not specify all of the below: age, weight and sex<br>No

	• "Female"<br>• "weight"	
Dosing	Analyze methods	Yes or No
Adverse events	Search terms<br>• "adverse"<br>• "Toler"<br>• "side"	Yes or No

Table S2. Methods to reduce bias.

Item	Search Strategy	Grading
Blinding	Search term<br>• "blind"	Yes<br>Partly: only blinded some of the experiments<br>No
Randomization	Search term<br>• "random"	Yes or No
Power calculation	Search term<br>• "Power"	Yes or No
Preregistration	Search terms<br>• "Prereg"<br>• "Regis"	Yes or No

References

1. Castle, J.; Blower, E.; Bundred, N.J.; Harvey, J.R.; Thachil, J.; Marshall, A.; Cox, K.; Cicconi, S.; Holcombe, C.; Palmieri, C. et al. Rivaroxaban compared to no treatment in ER-negative stage I–III early breast cancer patients (the TIP Trial): study protocol for a phase II preoperative window-of-opportunity study design randomised controlled trial. *Trials*. **2020**, *21*, 1–11.

© 2021 by the authors. Licensee MDPI, Basel, Switzerland. This article is an open access article distributed under the terms and conditions of the Creative Commons Attribution (CC BY) license (http://creativecommons.org/licenses/by/4.0/).
